# Expression profile of miRNAs computationally predicted to target PDL-1 in cervical tissues of different histology groups

**DOI:** 10.3389/fcell.2023.1101041

**Published:** 2023-02-24

**Authors:** Danai Leventakou, Alina-Roxani Gouloumi, Aris Spathis, Abraham Pouliakis, Nektarios Koufopoulos, Vassilios Pergialiotis, Peter Drakakis, Ioannis G. Panayiotides, Christine Kottaridi

**Affiliations:** ^1^ 2nd Department of Pathology, University General Hospital Attikon, School of Medicine, National and Kapodistrian University of Athens, Athens, Greece; ^2^ 1st Department of Obstetrics and Gynecology, Alexandra General Hospital, National and Kapodistrian University of Athens, Athens, Greece; ^3^ 3rd Department of Obstetrics and Gynecology, School of Medicine, University General Hospital Attikon, National and Kapodistrian University of Athens, Athens, Greece; ^4^ Department of Genetics, Development and Molecular Biology, School of Biology, Aristotle University of Thessaloniki, Thessaloniki, Greece

**Keywords:** miRNAs, cervical cancer, PDL-1, histology, gene expression profiling, HPV

## Abstract

**Background:** Human papilloma virus (HPV) is considered a successful pathogen as it has the ability to evade host immune responses and establish long-term persistent infection. It has been reported that programmed death ligand 1 (PDL-1) expression is correlated with HPV-positivity and is increased with lesion progression or tumor metastasis in cervical cancer. The expression of microRNAs (miRNAs) is often deregulated in cancer, and their potential targets are affected.

**Methods:** RNA was extracted from formalin-fixed paraffin-embedded (FFPE) cervical samples of different histological types, previously typed for the presence of HPV. A specific quantitative polymerase chain reaction (qPCR) protocol with SYBR Green was used to check for the expression of four miRNAs that were computationally predicted to target PDL-1.

**Results and conclusion:** hsa-miR-20a-5p and hsa-miR-106b-5p showed an expression increase with the severity of the lesions, while hsa-miR-125b-5p depicted a significant decrease in its expression in cancerous samples when compared to normal samples.

## 1 Introduction

Cervical cancer ranks third among all cancers in women worldwide. In 2018, 569847 women developed cervical cancer worldwide, with 311365 deaths being recorded. Cervical cancer is the second most common female cancer in women aged 15–44 years, with a large majority of the global burden occurring in the less developed regions (https://gco.iarc.fr/today accessed 1 April 2022). Since 1999, human papilloma virus (HPV) has been considered the main cause for cervical cancer (Ahmadzadeh et al., 2009). To date, more than 200 HPV types have been discovered, which are divided into cutaneous and mucosal HPVs based on the tissue they infect. The mucosal HPVs are clinically classified as “high-risk” and “low-risk” based on the propensity for malignant progression. HPV is considered a successful pathogen as it has the ability to evade host immune responses and establish long-term persistent infection.

Immune checkpoints are networks of inhibitory and stimulatory pathways that support immune response and sustain self-tolerance. Immune checkpoint mechanisms are frequently triggered in cancer to block the development of anti-tumor immune responses. Programmed cell death protein 1 (PD-1) is a 55-kDa transmembrane protein containing 288 amino acids with an extracellular N-terminal domain, a membrane-permeating domain, and a cytoplasmic tail (Ishida et al., 1992). Its ligand (PDL-1) is a member of the B7 family and is usually expressed by macrophages, some activated T cells, B cells, dendritic cells, and epithelial cells under inflammatory conditions (Sharpe et al., 2007). It is also expressed by tumor cells in order to avoid anti-tumor immune responses (Sanmamed and Chen, 2014). PD-1/PDL-1 launches self-tolerance by downregulating the immune system’s response and by suppressing T-cell inflammatory activity (Sanmamed and Chen, 2014).

PDL-1 expression is correlated with HPV-positivity and is increased with lesion progression or tumor metastasis in cervical cancer (Reddy et al., 2017; W. Yang et al., 2017). However, other studies (Brismar et al., 2010; Feng et al., 2018) report conflicting results, thus highlighting the necessity of further elucidation of the HPV role in the molecular mechanism through which the PDL-1 protein mediates tumor escape. miRNAs are non-coding RNAs with important functions in several biological processes, such as regulation of cell cycle, immune response, inflammation, and apoptosis. miRNAs attach to the 3′UTR of their target messenger RNA (mRNA) to inhibit its translation. Tumor-suppressor or oncogene expression can be disrupted by miRNA dysregulation, which is related to the pathogenesis of cancer, and many studies elucidate this relationship (Gurtner et al., 2016; Rupaimoole et al., 2016).

In this study, computationally predicted miRNAs that according to specific algorithm-based online tools bind to the 3’ UTR of PDL-1 mRNA were chosen, and their expression profile on formalin-fixed paraffin-embedded (FFPE) clinical samples of different histological categories was explored. The initial hypothesis was that if the studied miRNAs are capable of targeting PDL-1 mRNA and subsequently inhibiting its production, downregulation of studied miRNAs in high-grade lesions could be one of the reasons that PDL-1 is overexpressed, thus suggesting possible potential powerful candidates for therapeutic intervention.

## 2 Materials and methods

Histological sections were obtained from FFPE cervical biopsies of 115 non-pregnant Caucasian women aged between 20 and 81 years. Histological diagnoses included the following groups: normal, cervical intraepithelial neoplasia (CIN1, CIN2, and CIN3), squamous cell carcinoma (SCC), and adenocarcinomas. Details about the grade and stage of the cancer cases are presented in Table 1.

**TABLE 1:** Staging and grades of the cancer cases per cancer type. None of the cases was reported as metastatic.

Cancer type	Stage	Grade
SCC (22 cases)	pT1a1: 3 cases	Grade 1: 2 cases
	pT1a2: 5 cases	Grade 2: 16 cases
	pT1b1: 5 cases	Grade 3: 4 cases
	pT2a2: 5 cases	
	Unknown: 4 cases	
Adenocarcinoma (6 cases)	pT1a1: 2 cases	Grade 1: 2 cases
	pT2a2: 3 cases	Grade 2: 2 cases
	*In situ*: 1 case	Grade 3: 2 cases
Adenosquamous carcinoma (1 case)	pT2a2	Grade 3

All studied samples were previously genotyped for the presence of HPV using a commercially available real-time PCR assay kit (HPV Genotypes 14 Real-TM Quant, Sacace Biotechnologies, Como, Italy) after the DNA extraction with QIAamp DNA FFPE Tissue Kit (Qiagen GmbH) following the manufacturer’s instructions. miRNA and total RNA purification from formalin-fixed, paraffin-embedded tissue was performed using the miRNeasy FFPE Kit (Qiagen GmbH). The RNA’s quantity and quality were evaluated using QIAxpert (Qiagen GmbH). Reverse transcription was performed with the miScript II RT Kit (Qiagen GmbH), and for every reverse transcription reaction, the RNA concentration was adjusted to 500 ng, according to the kit manufacturer’s suggestions. The cDNA prepared was diluted properly, according to the protocol provided by the miScript II RT Kit (Qiagen GmbH), and 10–20 ng cDNA was used for the quantification of the studied miRNA and PDL-1 mRNA. For the real-time PCR quantification of mature miRNA, target-specific miScript Primer Assays (forward primers) and the miScript SYBR Green PCR Kit, which contains the miScript Universal Primer (reverse primer) and QuantiTect SYBR Green PCR Master Mix (Qiagen GmbH), were used. For the *PDL-1* gene expression analysis, QuantiTect Primer Assay (cat. number PPH21094A, Qiagen GmbH) was used for the real-time PCR amplification with SYBR Green. All cycling reactions were performed in duplicate on the QIAGEN Rotor-Gene Q (Corbett Rotor-Gene 6000) real-time PCR cycler.

The selection of the studied panel of miRNAs was based on the search in the following online tools: 1) DIANA TOOLS, 2) miRDB, and 3) TargetScanHuman (9–12). These computational miRNA target finders vary in the algorithm they use and were developed by analyzing thousands of miRNA–target interactions. The user may provide the miRNA name and search for the predicted gene target and *vice versa*. hsa-miR-106b-5p, hsa-miR-200a-3p, hsa-miR-125b-5p, and hsa-miR-20a-5p were suggested as possible miRNAs that target PDL-1 mRNA with the best cumulative scores.

Normalization of the miRNAs was performed using the geometric mean of all the benign samples, and the relative expression was calculated by using the 2^-ΔΔCt^ method. The stability of the normalization was verified using the geNorm macro, which displayed the lowest variation when using miR-23a and miR-191-5p. The two miRNAs were chosen based on the findings of Shen et al. (2011), where the combination of these two miRNA genes served as the optimal reference miRNAs that can be used as normalizers for miRNA RT-qPCR detection in clinical cervical tissues. Statistical analysis was performed in SPSS 25 for Windows and SAS 9.4 for Windows using non-parametric tests, since normality was not ensured (using the Shapiro–Wilk test). Specifically, we applied the Mann–Whitney *U*-test for the comparison of two groups or the Kruskal–Wallis test for the comparison of more than two histological groups. The identification of any potential relationship between women’s age and miRNA levels was performed using the Spearman correlation coefficient (r_s_). The statistical significance level was set to *p* < .05, and all tests were two-sided.

PDL-1 mRNA expression was analyzed in a similar fashion, with *ACTB* (actin beta) being used for normalization.

## 3 Results and discussion

The clinical samples with negative histology (*n* = 15) were HPV-negative, while all the remaining ones (*n* = 100) had at least one high-risk HPV type. The highly oncogenic HPV types 16 or 18 were detected in 64% of the HPV-positive samples (detailed results for the HPV subtypes identified in each histological category along with the number of cases with multiple infections, high-risk (HR) infections, and low-risk (LR) infections are presented in Table 2). In order to find a possible correlation between miRNA deregulation and highly oncogenic types HPV16 or HPV18 infection, miRNA expression analysis was performed, and no significant difference was revealed (data not shown).

**TABLE 2:** Outcomes of histological examination for the study population and HPV genotyping results. HPV subtypes in bold indicate high-risk subtypes. HR, high risk (number of women); LR, low risk (number of women).

		Number of cases according to identified HPV subtypes																										
Histological diagnosis	Number of cases (%)	**16**	**18**	**31**	**33**	**35**	**39**	**45**	**51**	**52**	**56**	**58**	**66**	**68**	6	42	43	53	61	62	70	81	82	83	84	Multiple infections	HR	LR
Negative	15 (13)																											
CIN1	30 (26.1)	10	5	3	4		2	4	7		5	2	6	1	3	1		5	2	1	1	1	1	1	1	19	30	12
CIN2	24 (20.9)	12	2	3	2	1			5	2	2	2	1	1	1	1	1	5	1				1			9	24	8
CIN3	17 (14.8)	12	1	2		2			1	3			1	1		1						1	2			6	17	3
SCC	22 (19.1)	17	1		1			3	1		1	2			1											4	22	1
Adenocarcinoma	6 (5.2)	5					1			1								1								2	6	1
Adenosquamous carcinoma	1 (0.9)							1										1								1	1	1
Total	115	56	9	8	7	3	3	8	14	6	8	6	8	3	5	3	1	12	3	1	1	2	4	1	1	41	100	26

**FIGURE 1:**
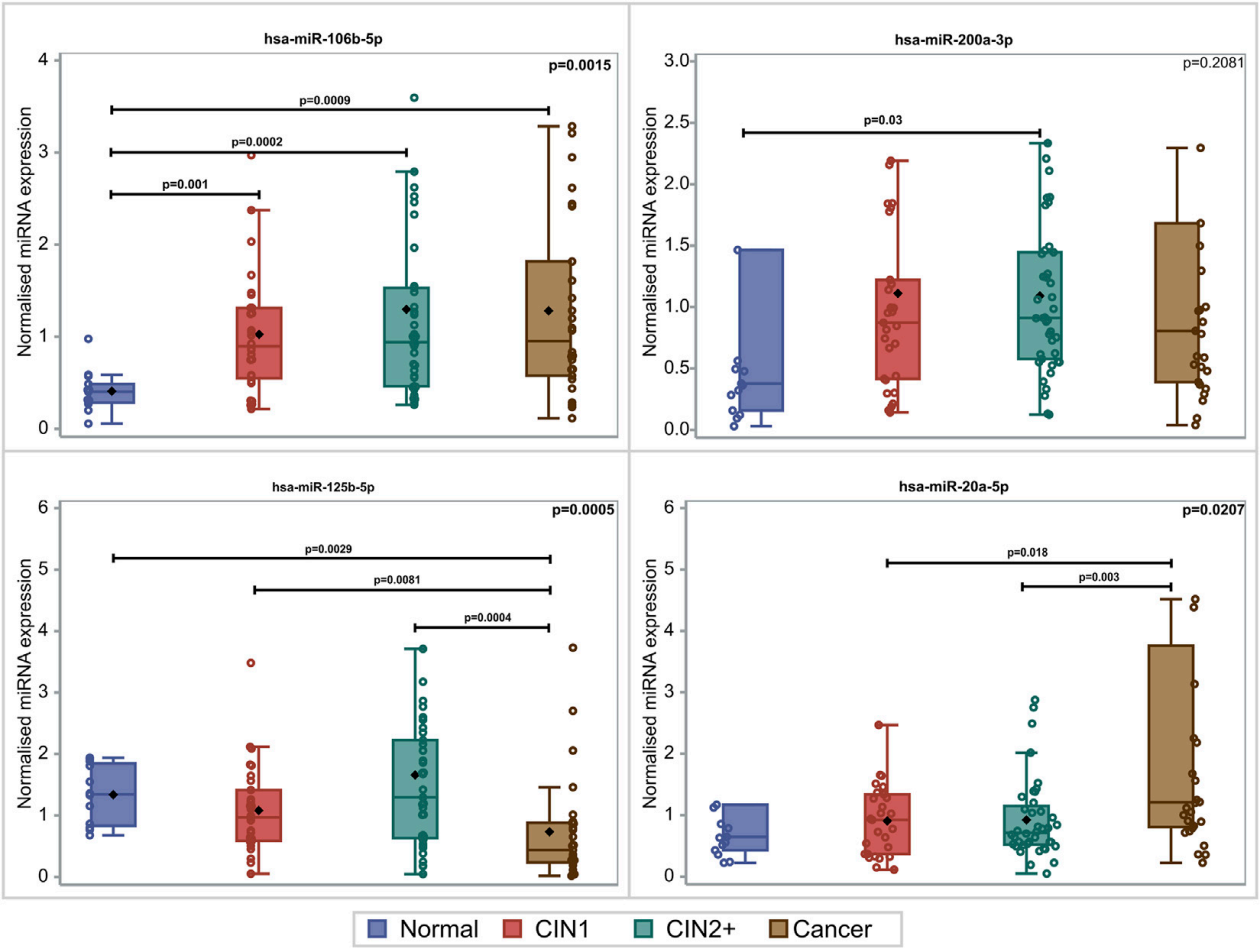
Box and whisker plots of the normalized miRNA levels in relation to the histological diagnosis. Box limits correspond to quartile 1 and quartile 3 values, the lines within the boxes correspond to the median values, and the rhomboid symbols correspond to the mean value. Whisker limits correspond to the minimum and maximum observation after outlier exclusion. Circles correspond to actual measurements. However, extreme measurements do not appear due to the scale of the vertical axis. *p*-values adjacent to box corners are the outcome of the Kruskal–Wallis test, and significant differences between pairs of histological groups (*via* the Mann–Whitney *U*-test) are presented *via* the horizontal lines and the relevant *p*-values.

PDL-1 mRNA expression was upregulated with the lesion progression. Specifically, the median and Q1–Q3 range level of expression for CIN1, CIN2+, and cancer cases were .45 (.22–2.44), 1.19 (.47–1.85), and 2.74 (.70–25.57), respectively (*p* = .0481). Mainly two miRNAs depicted statistically significant overexpression with lesion progression. For hsa-miR-20a-5p, overexpression between cancers and CIN1 (*p* = .018) as well as between cancers and CIN2+ (*p* = .003) was depicted. hsa-miR-106b-5p increased significantly in all lesions compared to normal samples (*p* = .009, *p* = .002, and *p* = .001), while no significant alteration of hsa-miR-200a-3p expression was identified between the different histological categories (*p* = .2081). The comparison of the expression profile of hsa-miR-125b-5p between normal, CIN1, and CIN2+ samples showed a slight decrease (*p* = .029, *p* = .0081, and *p* = .0004, respectively), indicating a downregulation while moving toward high-grade lesions (Figure 1). In this study, the combination of hsa-miR-23a-3p and hsa-miR-191-5p served as the optimal reference miRNAs that can be used as normalizers for miRNA RT-qPCR (real-time quantitative PCR) detection in cervical cancer tissues as previously published (Shen et al., 2011).

In relation to age, as expected, a significant difference (*p* < .0001) was observed among women with negative histology (median age: 46; Q1–Q3: 37–53 years), CIN1 (median: 31.5; Q1–Q3: 26–40), CIN2 and CIN3 combined (median: 31; Q1–Q3: 27–43 years), and cancer (median: 47; Q1–Q3: 42–59.5 years). Furthermore, the correlation coefficients of age and the miRNA expression were as follows: r_s_ = −.02 (*p* = .820) for hsa-miR-106b-5p, r_s_ = −.21 (*p* = .027) for hsa-miR-200a-3p, r_s_ = −.08 (*p* = .384) for hsa-miR-125b-5p, and r_s_ = .15 (*p* = .122) for hsa-miR-20a-5p. Notably, a statistically significant negative correlation was found between age and hsa-miR-106b-5p. However, since this is a weak correlation (r_s_ = −.21), it may not be attributed to some biological effects.

There are rather limited data to determine the role of miRNA expression in disease stage and grade, and thus, our analysis could not prove any significant difference for any of the studied miRNAs. However, for hsa-miR-200a-3p, there was a trend for higher values in T2 cases as compared to T1 (median: 1.64, Q1–Q3: .74–47.06 vs .54, Q1–Q3: .31–.92, respectively, *p* = .0726). In relation to tumor grading (four cases with grade 1, 18 cases with grade 2, and six cases with grade 3), it became impossible to confirm any significant difference for any of the miRNAs.

Since miRNAs can target multiple mRNAs, miR-based gene therapy can be an appealing approach to inhibit the *de novo* expression of oncoproteins, like PDL-1. Therefore, we conducted a bioinformatics-based search for miRNAs that possibly target PDL-1 with the purpose of an *in silico* suggestion of an miRNA that could serve as a negative regulator to suppress PDL-1, leading to mRNA degradation or translation repression.

According to our results, elevated levels of PDL-1 mRNA were recorded in squamous cell carcinomas and adenocarcinomas. In the study by Rischin et al. (2020), PDL-1 mRNA expression levels were analyzed based on data generated from The Cancer Genome Atlas (TCGA) Research Network (https://www.cancer.gov/tcga), and PDL-1 mRNA expression was found to be greater in squamous than in non-squamous cervical cancer samples. In our study, such a difference was not observed most probably due to the small size of squamous or non-squamous cervical cancer samples.

miR-106b-5p plays an important role in carcinogenesis as it acts as an oncomiR or as a tumor suppressor *via* regulating almost all cancer cell biological processes, including cell cycle, proliferation, apoptosis, differentiation, invasion, angiogenesis, drug resistance, and metastasis (C. Yang et al., 2020). In cervical cancer, a significant upregulation of miR-106b-5p has been confirmed, and it has been proposed that this upregulation modulates the expression of genes that play a crucial role in PI3K-Akt signaling, focal adhesion, and cancer (Yi et al., 2018). Previous studies showed that hsa-miR-20a-5p expression was markedly upregulated in cervical cancer tissues. Its overexpression could facilitate tumorigenesis and function as a potential diagnostic and prognostic biomarker in cervical cancer disease (Zhou et al., 2019). According to Gong et al. (2022), the bioinformatical prediction of hsa-miR-20a-5p binding to the 3′-untranslated region of PDL-1 was functionally validated and was correlated to an upregulation of PDL-1. Su et al. (2020) explored the biological function of hsa-miR-200a-3p in relation to cervical cancer, and after the inhibition of hsa-miR-200a-3p in HeLa cells, they concluded that this miRNA facilitates cervical cancer cell proliferation and activates the HIF-1α/VEGF (hypoxia-inducible factor 1 alpha/vascular endothelial growth factor) signaling pathway by targeting *EGLN1* gene (egl-9 family hypoxia-inducible factor 1). According to the literature, there is accumulating evidence verifying that the impact of hsa-miR-125b-5p on the development of cancers is extremely complex, and hsa-miR-125b-5p functions as both the oncogene and tumor suppressor in gastric cancer cells, bladder carcinoma, glioma hepatocellular carcinoma, and colorectal cancer (de Mello et al., 2021; Jesionek-Kupnicka et al., 2019; L. Sun et al., 2021; Tanaka et al., 2017; Wen et al., 2022). In cervical cancer tissues, hsa-miR-125b-5p expression was remarkably upregulated, and this overexpression was significantly related to a decrease in *HMGA1* gene (high mobility group AT-hook 1) expression, progression-free survival, overall survival, and prognosis (B. Sun et al., 2019). Wei et al. (2019) showed that PDL-1 directly interplays with HMGA1 to further activate the PI3K-Akt and MEK-ERK pathways in colorectal cancer. Specifically, the upregulation of PDL-1 increased the expression levels of HMGA1, a finding that could be worthy of investigation in cervical cancer.

In the present study, our effort was to evaluate whether the miRNAs computationally predicted to target PDL-1 have an expression profile that explains the correlation between these miRNAs and their target. The initial hypothesis was that if an miRNA revealed a downregulation in examined tissue samples, the observed downregulation could probably be in agreement with its target overexpression as a part of an underlying biological mechanism. Undeniably, a functional study would offer robust evidence on the true relationship between the evaluated miRNA and PDL-1. However, we aimed to preliminarily examine our cohort samples and suggest a possible correlation.

Evidence of such a possible correlation would be the decrease in the expression of the studied miRNAs in high-grade lesions, as PDL-1 is upregulated in cervical cancer. Obviously, although by means of bioinformatic tools, hsa-miR-20a-5p and hsa-miR-106b-5p are suggested as possible targets of PDL-1 mRNA. The findings of our study propose that these miRNAs should not be considered as such, but rather as oncomiRs, as this is justified by their overexpression in high-grade lesions. Although this finding is not in accordance with our expected hypothesis, it is a significant observation as recorded miRNA alterations could provide an attractive target for anticancer drug development.

Amongst the studied miRNAs in the present cohort, a slight decrease in the hsa-miR-125b-5p expression profile was depicted, thus proving a possible correlation between this computationally predicted miRNA and its target, which may be a part of a molecular pathway that increases PDL-1 expression. This imbalanced expression is another important observation that undoubtedly needs to be further clarified in a larger cohort so that the trend that is described here could be confirmed. A functional study to investigate whether PDL-1 constitutes a possible target of hsa-miR-125b-5p is of utmost necessity and is currently an object of ongoing research in our laboratory.

Recent research has revealed a strong correlation between miRNAs and the occurrence of different cancer types. With this short communication, we made an effort to clarify a possible correlation between miRNAs and PDL-1, intending to add in the rapidly gaining interest in the use of such molecules as alternative tools in the cervical cancer treatment strategy.
